# Exogean: a framework for annotating protein-coding genes in eukaryotic genomic DNA

**DOI:** 10.1186/gb-2006-7-s1-s7

**Published:** 2006-08-07

**Authors:** Sarah Djebali, Franck Delaplace, Hugues Roest Crollius

**Affiliations:** 1 Dyogen Lab, CNRS UMR8541, Ecole Normale Supérieure, 46 rue d'Ulm, 75005 Paris, France; 2 IBISC Lab, CNRS FRE2873, Université d'Evry Val d'Essonne, Genopole, 523 place des terrasses de l'Agora, 91000 Evry, France

## Abstract

**Background:**

Accurate and automatic gene identification in eukaryotic genomic DNA is more than ever of crucial importance to efficiently exploit the large volume of assembled genome sequences available to the community. Automatic methods have always been considered less reliable than human expertise. This is illustrated in the EGASP project, where reference annotations against which all automatic methods are measured are generated by human annotators and experimentally verified. We hypothesized that replicating the accuracy of human annotators in an automatic method could be achieved by formalizing the rules and decisions that they use, in a mathematical formalism.

**Results:**

We have developed Exogean, a flexible framework based on directed acyclic colored multigraphs (DACMs) that can represent biological objects (for example, mRNA, ESTs, protein alignments, exons) and relationships between them. Graphs are analyzed to process the information according to rules that replicate those used by human annotators. Simple individual starting objects given as input to Exogean are thus combined and synthesized into complex objects such as protein coding transcripts.

**Conclusion:**

We show here, in the context of the EGASP project, that Exogean is currently the method that best reproduces protein coding gene annotations from human experts, in terms of identifying at least one exact coding sequence per gene. We discuss current limitations of the method and several avenues for improvement.

## Background

Ideally, the process of annotating protein coding genes (hereby referred to as 'genes') in a region of genomic DNA involves locating the exact external and internal boundaries of all the genes it includes and, for each, finding all the possible transcript variants. In practice, achieving this is very difficult in eukaryotic genomes for many reasons. First, eukaryotic genes are generally composed of a succession of exons and introns, which makes their structure complex and highly variable. Second, genes cover only a small fraction of eukaryotic genomes (30% in mammals) and exons cover an even lower fraction (1% to 2% in mammals). Third, some eukaryotic genomes contain many pseudogenes, which are non-functional copies of genes sometimes nested within genes and with similar compositions. Finally, each gene may give rise to many different transcripts, often with minor variations, a mechanism that modulates the function or the spatial or temporal availability of the corresponding protein. Despite these difficulties, precise gene annotation is crucial for biomedical research: it is a basic requirement to link genotype and phenotypes in human and model species and generally to focus the work of biologists and bio-informaticians on an essential functional part of the genome. Forty eukaryotic genome sequences have now been completed and each is commonly tens of millions or even billions of nucleotides long: annotating genes in this massive amount of data undoubtedly requires mathematical models.

Mathematical models have been proposed to automatically locate genes in genomic DNA, either by similarity to expressed or evolutionary conserved sequences, or by capturing our current biological understanding of genes in statistical algorithms, or a combination of these methods. Despite tremendous advances, automatic gene annotations are still considered predictions that require validation by human experts, particularly when expensive and time consuming experimental work will be based upon them. This paradigm is exemplified in the ENCODE Genome Annotation Assessment Project (EGASP) competition [1], where the reference against which all the automatic methods are measured is a set of annotations experimentally verified and manually curated by human experts. Such high quality reference gene annotations (also including those collated in the Vega repository [2]) are generated by humans based on a number of resources: cross-species sequence alignments, mRNA sequences, *ab initio *predictions, and so on. Generally, with the aid of sophisticated annotation and curation software tools, these resources are reviewed on a gene-by-gene basis using strict rules rooted in a deep knowledge of both the data at hand and the biology associated with gene expression (transcription, splicing, translation, and so on).

Based on these observations, we were interested in designing an automatic annotation method that explicitly establishes the same relationships between biological objects, and applies the same rules, as human experts. In computer science, some such rules can be assimilated to heuristics of the type '*if (X) *then *(Y) *else *(Z)*'. The automatic annotation of protein coding genes may, therefore, appear deceivingly simple and be reduced to coding the rules extracted from biological expertise into a set of heuristics and to applying them to the experimental evidence. In the field of gene annotation, however, encoding human expertise is rather a problem of untangling the body of evidence that experts build to elect a sequence to the status of protein coding gene. This body of evidence can be viewed as a complex network of relationships between DNA, mRNA and protein sequences. These relationships are difficult to formalize because biological knowledge cannot (yet) be assimilated to a structured list of observations based on a controlled vocabulary. It is instead a rich and heterogeneous set of often unconnected observations. It is also in constant evolution and may, therefore, vary from one set of experts to another. It does not always follow strict logical rules and instead may rely on arbitrary variables. In the context of gene annotation, the latter is often a consequence of a lack of knowledge on specific aspects of gene structure and biology. For instance, a transcript with an annotated coding region of less than 100 amino acids is often considered too short and not classified as coding for a protein.

Our first objective was, therefore, to design a formal framework within which rules and biological objects may be represented and manipulated using computers to produce gene annotations. We then identified a number of rules and biological objects used by human annotators and integrated them into the framework. The resulting software tool is called Exogean for EXpert On GEne Annotation. We believe that this strategy is currently the only possibility to approach the level of completeness and accuracy reached by human experts. Here we show, in the context of the EGASP competition of the ENCODE project, that Exogean already performs better than any other automatic method in identifying at least one exact coding sequence per gene.

## Results

### The Exogean method

Human annotators manipulate and integrate information stemming from multiple heterogeneous sources (for example, *ab initio *predictions, mRNA and expressed sequence tag (EST) alignments, protein alignments). Each source has specific properties and is thus treated with specific rules. For instance, mRNA sequences from the same species that is being annotated should align to the genome with high similarity (98% to 100%) while protein sequences from a different species typically align less perfectly owing to base substitutions, insertions or deletions during evolution. Hence, mRNA alignments are dealt with using more stringent rules and are given more importance because they can be aligned perfectly, while protein alignments are typically treated with more caution. The different sources of data will, therefore, be represented differently, and will be processed by different heuristics.

To develop a flexible yet formal framework, we decomposed human expertise into heuristics of two independent types: the establishment of relationships between objects on the one hand; and the action of connecting and merging the objects based on these relationships on the other hand.

This independence between relationships and actions provides the flexibility required to solve a number of difficulties: heuristics change over time - the system must be able to easily adapt to these changes by modifying, adding or deleting heuristics; heuristics are applied to different sources of information (for example, different types of sequence alignment) - the system must be able to handle heterogeneous sources; heuristics are themselves of different types, whether they deal for instance with structural concepts (properties of aligned sequences) or on prioritizing sources - the system must be able to handle heterogeneous heuristics.

**Figure 1:**
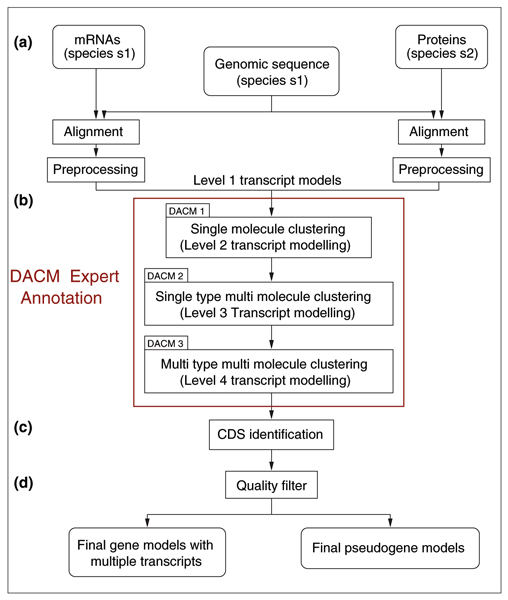
Overview of the Exogean methodology. Exogean annotations are based on protein and/or mRNA alignments. Protein sequences come from a different species than that of the DNA to be annotated (for example, mouse protein to annotate human genes), while the mRNAs originate from tissues of the species being annotated. **(a) **These alignments are first processed to remove artifacts using heuristics. **(b) **The core of the method is the directed acyclic multigraph (DACM) component, which builds transcripts of increasing complexity in three iterative steps using a second set of heuristics. **(c) **The transcripts are then examined to identify a complete or a partial coding sequence (CDS), and **(d) **a last filter applies a last set of rules to remove pseudogenes.

**Figure 2:**
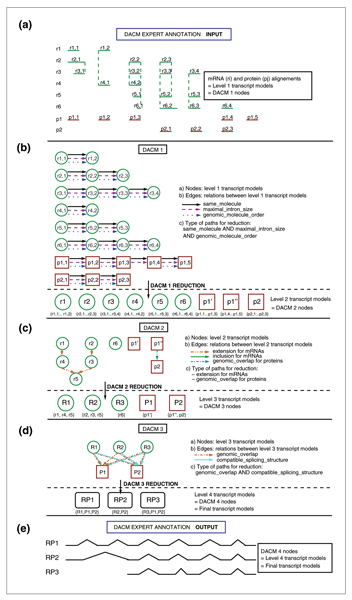
Example of transcript modeling from a set of protein and mRNA alignments using DACMs. **(a) **The DACM input are mRNA (r1...r6) and protein (p1, p2) sequences that have been aligned to a genomic sequence S. The individual local alignments are each a level 1 transcript model (L1TMs)and constitute the nodes of a graph DACM1. **(b) **This graph has three possible directed edges: same_molecule, maximal_intron_size, and genomic_molecule_order. Each corresponds to a different relationship that connects two nodes if they respectively: are alignments produced by the same mRNA or same protein; are separated by a distance smaller than a user defined threshold (for example, 75 kilobases); and are collinear on the molecule of origin (mRNA or protein) and the genomic DNA. There are nine maximal paths along the three combined edges, which reduce DACM1 into the nine nodes (r1 to r6 and p1', p1", p2) of a graph DACM2, each representing a level 2 transcript model (L2TM). Note that the reduction of DACM1 splits nodes p1,1 to p1,5 into two DACM2 nodes (p1' and p1) because of the absenceof a genomic_molecule_order edge between p1,3 and p1,4. **(c) **DACM2 has three possible edges, inclusion, extension (for mRNAs) and genomic_overlap (for proteins), which respectively connect two nodes if: they overlap and their overlapping introns are identical; they overlap and their overlapping introns are identical but the second node also extends the first in 3'; and the span of the two nodes have overlapping genomic coordinates. The reduction follows either the 'extension' rule for mRNAs edges or the genomic_overlap protein edge and produces here the five nodes of graph DACM3 (mRNA nodes R1 to R3 and protein nodes P1 and P2), which represent level 3 transcript models (L3TMs). **(d) **DACM3 has two possible edges, genomic_overlap and compatible_splicing_structure, whichconnect (combines) protein and mRNA transcript models if they respectively have overlapping genomic coordinates and if the protein transcript model does not have any exons in introns of the mRNA transcript model. To reduce the graph, Exogean first identifies the path that contains both edges and from these, the reduction consists in grouping all nodes that are connectedto the same RNA node. This generates the three nodes of a graph DACM4 (RP1 to RP3), which represent level 4 transcript models (L4TMs). These L4TMs arethe final transcript models generated by the DACM expert annotation. **(e) **Graphical representation of the DACM expert annotation output: the final transcript models RP1 to RP3 are represented on the genomic sequence S. No information has been lost during the three graph reductions. Note that transcript models produced by the DACM component of Exogean are not yet final, and will be further examined and potentially extended when looking for splicing and start/stop signals.

In Exogean (Figures 1 and 2), transcripts are built from relatively simple objects (sequence alignments) into more complex structures. Throughout the manuscript, therefore, the term transcript model will designate transcripts at any level of complexity, from basic sequence alignments to the final structure that represents the predicted functional mRNA (Figure 2). Following this we define that heuristics of the type 'relationship' will always be established between two transcript models of the same level of complexity. The fact that relationships may be directional (can be represented as an arrow between two objects) greatly simplifies the actions (see below) and is based on the directionality of the DNA molecule and, hence, of transcription itself. Relationships are thus directed by default, unless otherwise specified.

To represent transcript models and relationships, and to apply actions on the former using the latter, we use directed acyclic coloured multigraphs (DACMs; Figures 1 and 2). In such graphs, nodes are transcript models and multiple edges between nodes are the relationships. In its current version, Exogean uses three DACMs, each with increasingly complex and accurate transcript models (nodes) and different relationships (edges). While DACM1 is built from the original sources of information given to Exogean (mRNA and/or protein alignments), its output will be the basis of DACM2, and DACM3 will be built from the output of DACM2. To proceed from one DACM to the next, Exogean performs a graph reduction. The first step in reducing a graph involves the definition of a set of relationships (edges) that will represent a certain path. Then Exogean finds all the paths of maximal length in the DACM, which results in combining the different nodes located on each of these paths. The nodes collected along a maximal path together form a more complex object (transcript model) that will be a new node ready for processing in the next DACM. Edges are then built between these nodes, and this constitutes a new DACM that, in turn, can be reduced. In summary, Exogean automates the annotation protocol followed by human experts by iteratively building edges (making relationships) and subsequently reducing DACMs (taking actions based on these relationships). Before and after the three core DACMs, Exogean also applies heuristics to respectively prepare the data for transcript modeling, and to identify coding sequence (CDS) within transcripts (Figure 1).

### The EGASP assessment

Exogean is one of 20 automatic methods that were compared in the EGASP project [1] (see Materials and methods). In brief, each method predicted protein coding transcripts in 31 regions of the human genome totaling about 21 Mb. Independently, a group of experts (the Havana group at the Sanger Institute [3]) annotated the same regions using manual curation and experimental validation and identified 296 genes that were considered as reference against which all the automatic methods were compared. We refer to this set of genes as the GENCODE annotations [4]. The comparison between GENCODE annotations and Exogean predictions is summarized in Table 1. Except for the DNA sequence itself, the only source of information used by Exogean to predict transcripts were human mRNA and mouse protein sequence alignments (see Materials and methods). Exogean predictions are evaluated both if they overlap and if they exactly match a GENCODE annotation (see Materials and methods).

#### Overlapping predictions

Two standard measures to evaluate the accuracy of predictions against a reference are sensitivity (percentage of annotations identified) and specificity (percentage of predictions that identify an annotation). In the overlap evaluation, Exogean consistently detects GENCODE coding nucleotides, exons, transcripts and CDS with more than 80% sensitivity and 94% specificity. Of particular interest is the identification of transcripts and genes, where Exogean predicts less than 3% false positives (15 transcripts out of 513 predicted) corresponding to 8 genes that do not overlap a Havana gene. We investigated in more details the reasons why Exogean predicted these sequences as genes, since some may potentially represent new CDS. Two predictions correspond to retro-transposable elements (one L1 and one LTR) that are both supported by at least one mRNA aligned at these positions. One prediction corresponds to the H19 maternally imprinted non-coding RNA on chromosome 11, where Exogean nevertheless predicts a 356 amino acid protein sequence across its 5 exons spanning more than 50% of the length of the RNA. Another prediction is a GENCODE putative gene directly downstream of H19 supported by a single human placental mRNA that, upon manual inspection, displays no pseudogene characteristics but shows no similarity to any known protein. GENCODE 'putative' genes are not considered *bona fide *coding transcripts in this evaluation, and it is thus considered an incorrect Exogean prediction. Finally, four predictions correspond to clear cases of pseudogenes that Exogean did not identify as such. In conclusion, across the 31 Encode regions tested, Exogean predicts only six true false positive genes that do not overlap GENCODE annotations, and two neighboring genes that are expressed in the form of RNAs but are either putative CDS or known non-coding functional RNA.

Conversely, Exogean misses 53 GENCODE protein-coding genes out of 296 (18%). Examination of each case revealed eight possible causes, listed in Table 2. The most prominent reason for which Exogean fails to predict a gene overlapping a GENCODE annotation is when such genes are organized in clusters (33 genes missed). In these situations, homologous mouse proteins invariably produce alignments to most genes in the cluster because they all share a high sequence similarity. If one such mouse protein bridges two or more neighboring genes by producing alignments that are contiguous both in the protein sequence and in the genomic DNA, this contiguity will not be eliminated by Exogean provided it continues to comply with the other rules. Consequently, Exogean defined transcripts spanning the entire cluster and the CDS found in each prediction only covers one or perhaps two GENCODE annotations, resulting in most genes in the cluster being missed. Most cases (26 out of 33) concern the Encode region ENm009, which contains an olfactory receptor gene cluster. Clearly, the rules currently implemented in Exogean for exploiting protein alignment need revisiting to address such cases, which theoretically should not pose a major problem and thus provide a rich avenue for improvement. The other causes for false negative predictions each concern fewer cases (between 1 and 8). For instance, Havana annotated 42 transcripts in 29 genes with a CDS smaller than 300 nucleotides. Exogean currently does not predict CDS that would produce a protein with less than 100 amino acids, which prevented predictions overlapping eight of these GENCODE genes. Three GENCODE genes were not predicted because they are interrupted by the limits of the Encode region. Since we filtered out as potentially unreliable any evidence that was truncated by the boundaries of Encode regions, Exogean was unable to predict transcripts in these genes. The remaining eight GENCODE genes that were not predicted by Exogean are due to rules implemented in the program that are slightly too stringent, resulting either in the elimination of some evidence or in the inability to identify rare forms of intron donor/acceptor sites. These rules can thus probably be refined further.

#### Exactly matching predictions

**Figure 3:**
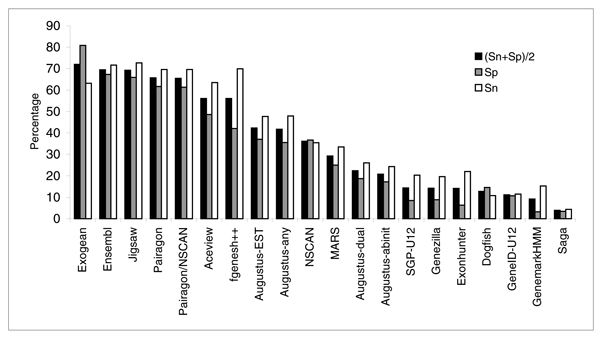
Exogean outperforms all other automatic methods confronted during EGASP. Histogram of the performances in terms of specificity (Sp, grey), sensitivity (Sn, white), and average between specificity and sensitivity ((Sn+Sn)/2, black) for all methods designed to predict protein-coding sequences in the EGASP competition. The values were provided by the EGASP organizers, and are based on predictions matching at least one coding sequence (CDS) exactly for each Havana annotated gene (known or novel). Themethods are ranked left to right from best to worse average between Sn and Sp.

The difficulty in automatically annotating protein-coding genes in eukaryotic DNA lies not so much in identifying predictions that at least partially overlap the coding sequence of each real gene but rather in identifying the precise positions of the coding sequence of every transcript, that is, the start codon, all the internal exon boundaries if they exist, and the stop codon. In designing Exogean, we have focused on maintaining a high specificity in order to obtain a strong and reliable baseline annotation, with as few compromises as possible on sensitivity. This is reflected in Exogean's specificity in exact gene CDS predictions (Table 1), which is higher than any other method by a large margin: no other method shows more than 70% specificity while Exogean shows more than 80% specificity (Figure 3). Does this come at the cost of a low sensitivity? A group of seven methods including Exogean show a distinctly better sensitivity (between 63% and 73%) than all the others (below 50%). Exogean's sensitivity (63%) is not, therefore, notably affected by the quest for a high specificity. In fact, four GENCODE genes are uniquely identified by Exogean (supported by both mouse proteins and human mRNAs) and by no other method from the same category. Altogether, based on the average between specificity and sensitivity (a standard measure to compare different methods [5,6]) for exact gene CDS predictions, Exogean comes in first position when ranking all the methods that participated in the EGASP competition (Figure 3).

**Figure 4:**
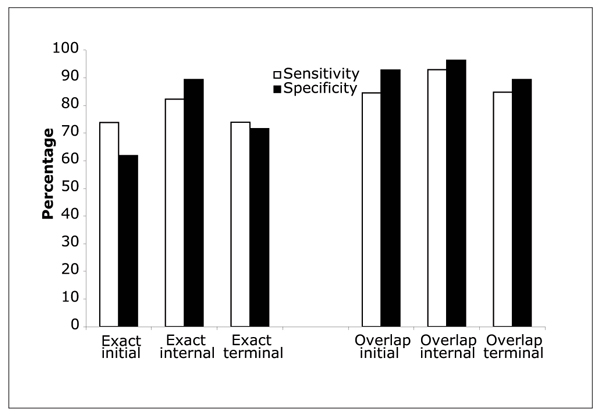
GENCODE and Exogean agree more often on exact boundaries of internal rather than external exons. Histogram of Exogean sensitivity and specificity in identifying the exact boundaries (left) and overlapping boundaries (right) of Havana initial, internal and terminal exons. While Exogean more or less predicts overlapping exons with similar specificities for initial, internal and terminal exons, this is not the case for exactly matching exons: Exogean internal exons are much more specific (89%) than external ones (62% and 72%).

Like Havana, Exogean is able to predict several alternative transcripts per gene: Exogean and Havana identify on average 2.34 and 2.19 isoforms per gene, respectively. Interestingly, while its sensitivity for detecting transcripts is the second highest across all methods, Exogean does not predict exactly matching transcripts with the same high specificity as for genes (Table 1). To explain this apparent contradiction, we were interested to see if a specific category of GENCODE transcripts is better predicted than others. Indeed, out of 267 exact transcripts predicted by Exogean, a remarkable 266 correspond to GENCODE transcripts that are complete, that is, where a start and a stop codon have been found. The single remaining exact Exogean transcript matches one of the 194 incomplete GENCODE transcripts. This result has two important consequences: the first is the conclusion that Exogean reproduces GENCODE annotations much better when the latter are complete transcripts. The second is the suggestion that a fraction of the complete Exogean transcripts overlapping incomplete GENCODE transcripts, and thus not showing exact matches, might be correct. Indeed, there are 23 GENCODE genes that only include incomplete transcripts, and complete Exogean transcripts overlap 11 of these 23 genes. It is thus not excluded that Exogean is able to completely and correctly annotate genes that were partially annotated by Havana. If this was the case, one would expect discrepancies between Exogean and GENCODE transcripts to occur more often at the end of transcripts, where arbitrary end points are more frequent. Figure 4 shows indeed that initial and terminal exons are less well identified exactly by Exogean than internal ones, although Exogean does overlap these external exons with the same sensitivity as internal ones.

An important factor that likely explains why Exogean transcripts are exact in complete GENCODE transcripts but not in partial ones is that Exogean only uses human mRNA and mouse protein alignments, while Havana also includes human ESTs among other additional sources. When a GENCODE annotation is only supported by ESTs, then Exogean will often predict a different transcript or no transcript at all, depending on the conservation of the corresponding protein in mouse. This affects mainly incomplete GENCODE transcripts because ESTs typically cover only parts of complete transcripts of a given gene. Conversely, if mRNA evidence exists for a gene, then Exogean and Havana will both use it and are thus more likely to predict the same corresponding transcript, which is more likely to be complete because mRNAs tend to cover the entire length of transcripts. We are currently formulating heuristics that will also allow Exogean to take ESTs into account.

**Figure 5:**
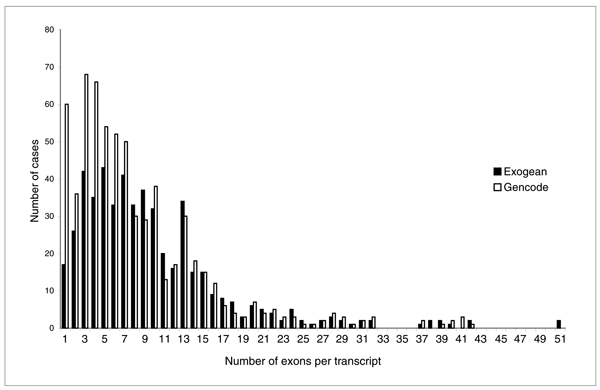
Histogram of the number of Exogean and Havana predicted transcripts as a function of their respective number of exons. For transcripts with large numbers of exons (more than 9), Exogean and Havana predict remarkably similar numbers of transcripts.

**Figure 6:**
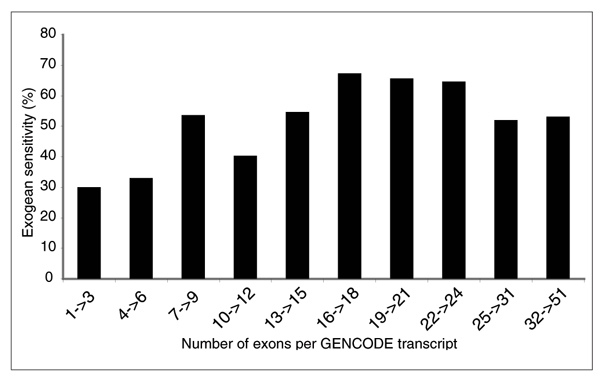
Histogram of Exogean's sensitivity in predicting exact GENCODE transcripts as a function of the number of exons in the latter. Clearly, GENCODE transcripts with larger numbers of exons are better identified than smaller transcripts.

Finally, Exogean predictions show the highest average number of exons per transcript (9.8) compared to Havana (8.28) and all other methods (below 8.6). One factor contributing to this high figure is that Exogean predicts fewer transcripts with few exons than Havana (Figure 5). In contrast, Havana and Exogean predict a remarkably similar number of transcripts with many exons (more than 9 exons) and this is accompanied by a higher sensitivity in correct predictions (Figure 6) for these particular transcripts. Here also, the different sources of evidence used by the two strategies probably explain these observations: transcripts with many exons are more likely to be predicted based on mRNA alignments, while shorter transcripts probably reflect more EST-based alignments, simply because ESTs are generally shorter than mRNAs.

#### An improved version of Exogean (post-EGASP)

Since the EGASP experiment, we have addressed many of the limitations described above in a new version of Exogean. Major areas of improvement have focused on refining the rules to untangle protein alignments in clusters of paralogous genes, and in the definition of the CDS of transcripts. These rules have a direct positive impact on sensitivity, with very little consequence on specificity; sensitivity in exact GENCODE CDS identification increases to 72.64% and specificity remains essentially stable at 79.30%. The average between these two measures is 75.97%, which demonstrates a substantial improvement over the version used in EGASP (72.00%). Using this version, we tested the influence of the nature of the information provided to Exogean. Indeed, human mRNAs generally provide a high specificity and precise exon boundaries but only cover a subset of genes, while each mouse protein tends to identify a broad spectrum of genes in human (paralogs), albeit with fuzzy boundaries. The complementarity of these two sources of information is confirmed when each is used individually and then in combination. Human mRNAs alone provide very specific predictions for a substantial fraction of GENCODE CDSs (sensitivity and specificity for exact CDS prediction are 64.86% and 82.11%, respectively) and mouse proteins provide little sensitivity and little specificity on exact CDS predictions (17.23% and 50.00%, respectively). But combining both sources yields the performances described above for the new version of Exogean, with a sharp increase in sensitivity and a minor decrease in specificity compared to the use of human mRNAs only. Of note, the sensitivity of Exogean predictions increases by 8% when mouse proteins are added to human mRNAs. The reason stems from a rule that forbids the use of single exon transcript models based solely on human mRNAs, whereas many such genes are recovered by mouse proteins.

## Discussion

Conclusions from previous studies aiming at comparing automatic annotation methods in eukaryotic genomes have often been limited by the availability of a large and reliable reference dataset. In this respect, the EGASP assessment project has been a unique opportunity to rigorously measure how well current strategies replicate meticulous and detailed protein coding gene annotations on a large and varied set of genomic regions in a blind test [1] (see Materials and methods). A commonly accepted standard for annotating genes is to consider that at least one coding transcript must be entirely and exactly identified [7]. Using this measure, Exogean is the method that currently best replicates reference annotations out of 20 methods tested in EGASP. In particular, Exogean is the most specific by a large measure (12% more than the next best), which reflects our initial objective when designing the method. In addition, out of all methods with good performances, Exogean is also the most consistent across the 31 ENCODE regions [1]. This suggests that Exogean would be the most likely method to reproduce its performances on a different set of human genomic regions. Because 8 out of 10 genes annotated by Exogean are correct, one possible use of Exogean is to accelerate annotations by human experts, especially since the methodology behind Exogean intuitively follows the same logic. To assist in this task, Exogean generates (in addition to the positions of transcripts and their sequences) information on each predicted gene and transcript that summarizes their structure, the evidence used, the problems and conflicts encountered and the solutions applied. Human experts may continue from there and use additional rules, resources and experiments to correct or confirm the automatic predictions.

While Exogean is specific, its sensitivity could be improved in several ways. First, the annotations that we produced for the EGASP assessment relied solely on two sources of alignments: human mRNA sequences and proteins predicted in the mouse genome. It is thus not unexpected that other participating methods that rely on a wider range of resources (human ESTs, mRNAs from other species, conserved genomic DNA) identify more genes, and we are currently designing rules to integrate some of these resources as well. We also show that current rules designed to exploit mouse protein alignments fail when human genes are in clusters, such as the olfactory receptor gene cluster. The EGASP experience was extremely useful in helping to uncover such limitations, many of which have been addressed in a new version of Exogean.

Automatic annotation methods have traditionally used statistical models to capture properties of genes and annotate them in genomic DNA, either alone [8,9] or in combination with evidence from other sources [10-13]. Exogean departs from these approaches in that it only relies on rules extracted from human expertise, and as such does not need to train on a set of known genes to 'learn' their statistical properties. Directed acyclic graphs (DAGs), the component used in the Exogean strategy to store and manipulate the information, have already been used in the context of gene annotation, albeit differently. The program AIR [14] uses DAGs where exons are nodes and edges are introns. ESTGenes [15] uses similar DAGs to Exogean but with a unique edge between nodes, whereas multiple edges are used in Exogean's multigraphs. One advantage of using DAGs as in ESTGenes or Exogean is the strict independence that can be maintained between the data and the heuristics applied to the data.

Translating human expertise for gene annotation into a computational framework could be generalized if an appropriate language was developed. The formalization that is at the core of Exogean, namely the DACM algorithm, can be seen as a natural starting point for developing such a language. If successful, this approach could lead to a more general and expressive method to integrate any rule that biologists use to synthesize information about biological objects in order to create more complex objects. Such approaches could potentially be of great use in the future.

## Materials and methods

### The Exogean software

Exogean is written in Ocaml [16]. Precompiled executables are available for several platforms [17]. Exogean is currently able to annotate eukaryote protein coding genes based on alignments with proteins from a different species and/or mRNAs from the same species. Input files with the alignments must be provided in one of several possible formats (psl, gff or exf, the latter being a simple format developed for Exogean). For the EGASP assessment, Exogean used proteins from the mouse International Protein Index database (March 2005 version containing 42,799 protein sequences) and human mRNA from EMBL (March 2005 release, containing 213,695 mRNA sequences) aligned by BLAT [18]. If protein alignments are used, a fasta formatted file with the protein sequences must also be provided. Finally, a configuration file is required where a large number of parameters pertaining to the rules used by Exogean are specified. Given that the alignments are computed, Exogean is fast since the entire human genome is annotated in approximately 100 minutes on a single 3 GHz processor with 1 Gb memory. A formal description of the Exogean method will be described elsewhere.

### The EGASP assessment

To place in their context the results described here, we briefly summarize the conditions of EGASP [1]. EGASP was organized by the GENCODE [19] group of the ENCODE project [20] and the aims were twofold: to evaluate how well automatic methods are able to reproduce manual and experimental gene annotation of the human genome; and to assess how complete our current knowledge is of the gene content of the human genome. A set of 31 regions of the human genome from the ENCODE project totaling 21.5 Mb of DNA were used by 14 groups to predict protein coding genes. After submitting the predictions, a workshop [21] was organized to confront the prediction and the annotations from the Havana group [3] at the Sanger Institute. The Havana group annotates genes by combining information from a variety of sources using human expertise and experimental validations (designated here as the GENCODE annotations [4]). All the figures for sensitivity and specificity used for Table 1 and Figure 3 are those provided by the EGASP organizers based on these comparisons. When necessary (Table 1, Figures 4 and 6, and text) additional results for Exogean were computed using the Eval software [22], kindly provided by the organizers. In the overlap mode, any GENCODE annotation with boundaries that overlap an Exogean prediction is counted as true positive.

## Figures and Tables

**Table 1:** Exogean performances in identifying GENCODE coding sequences

Measure	Sensitivity (%)	Specificity (%)	Exogean correct	Exogean wrong	GENCODE missed	GENCODE total
Overlap						
Nucleotides	84.18	94.33	371,369	22,301	69,791	441,160
Exons	90.12	94.94	2,495	133	273	2764
Transcripts	89.37	97.08	498	15	151	649
Genes	82.09	96.35	211	8	53	296
Exact						
Nucleotides	84.18	94.33	371,369	22,301	69,791	441,160
Exons	79.34	83.45	2,193	435	571	2764
Transcripts	42.53	52.44	267	237	373	649
Genes	63.18	80.82	187	32	109	296

**Table 2:** Diagnostic for GENCODE genes missed by Exogean

Class of diagnostic	Number of cases	Description of the cause
1	33	Proteins matching multiple neighboring genes in clusters
2	8	GENCODE CDS size below 300 nucleotides
3	3	GENCODE CDS interrupted by the boundaries of the Encode region
4	2	Insufficient evidence to predict the gene
5	3	Protein evidence eliminated by Exogean
6	1	mRNA evidence eliminated by Exogean
7	2	Intron donor/acceptor sites not accepted by Exogean
8	1	mRNA evidence was misaligned with genomic DNA
